# Onyx^TM^Gel or Coil versus Hydrogel as Embolic Agents in Endovascular Applications: Review of the Literature and Case Series

**DOI:** 10.3390/gels10050312

**Published:** 2024-05-02

**Authors:** Paolo Perri, Giuseppe Sena, Paolo Piro, Tommaso De Bartolo, Stefania Galassi, Davide Costa, Raffaele Serra

**Affiliations:** 1 Department of Vascular and Endovascular Surgery, Annunziata Hospital, 1 Via Migliori, 87100 Cosenza, Italy; p.perri@aocs.it (P.P.); p.piro@aocs.it (P.P.); 2 Department of Vascular Surgery, “Pugliese-Ciaccio” Hospital, 88100 Catanzaro, Italy; giuseppe.sena@aocz.it; 3 Departement of Interventional Radiology, Annunziata Hospital, 1 Via Migliori, 87100 Cosenza, Italy; t.debartolo@aocs.it (T.D.B.); s.galassi@aocs.it (S.G.); 4 Interuniversity Center of Phlebolymphology (CIFL), Magna Graecia University of Catanzaro, 88100 Catanzaro, Italy; davide.costa@unicz.it; 5 Department of Medical and Surgical Sciences, Magna Graecia University of Catanzaro, 88100 Catanzaro, Italy

**Keywords:** embolic agent, hydrogel, coil, endovascular application

## Abstract

This review focuses on the use of conventional gel or coil and “new” generation hydrogel used as an embolic agent in endovascular applications. In general, embolic agents have deep or multidistrict vascular penetration properties as they ensure complete occlusion of vessels by exploiting the patient’s coagulation system, which recognises them as substances foreign to the body, thus triggering the coagulation cascade. This is why they are widely used in the treatment of endovascular corrections (EV repair), arteriovenous malformations (AVM), endoleaks (E), visceral aneurysms or pseudo-aneurysms, and embolisation of pre-surgical or post-surgical (iatrogenic) lesions. Conventional gels such as Onyx or coils are now commercially available, both of which are frequently used in endovascular interventional procedures, as they are minimally invasive and have numerous advantages over conventional open repair (OR) surgery. Recently, these agents have been modified and optimised to develop new embolic substances in the form of hydrogels based on alginate, chitosan, fibroin and other polymers to ensure embolisation through phase transition phenomena. The main aim of this work was to expand on the data already known in the literature concerning the application of these devices in the endovascular field, focusing on the advantages, disadvantages and safety profiles of conventional and innovative embolic agents and also through some clinical cases reported. The clinical case series concerns the correction and exclusion of endoleak type I or type II appeared after an endovascular procedure of exclusion of aneurysmal abdominal aortic (EVAR) with a coil (coil penumbra released by a LANTERN microcatheter), the exclusion of renal arterial malformation (MAV) with a coil (penumbra coil released by a LANTERN microcatheter) and the correction of endoleak through the application of Onyx 18 in the arteries where sealing by the endoprosthesis was not guaranteed.

## 1. Introduction

In recent years, endovascular interventional procedures aimed at transcatheter arterial embolisation have largely replaced traditional open repair surgery as they are minimally invasive and have numerous vantages [1]. Due to its manoeuvrability, low cost, few complications and high efficacy, endovascular embolisation is considered a practical and effective method for the treatment of various vascular pathologies, such as endovascular corrections (EV repair) [2], arteriovenous malformations (AVM) [3], endoleaks (E) [4,5], aneurysms or pseudo-aneurysms [6,7], and haemorrhages [8].

Embolising agents are defined as particles or fluids that can be released into the bloodstream to mechanically and/or biologically occlude the target vessel [9]. Commercially, they are available as solids, liquids and suspensions [10]. Solid embolic agents include coils and metal plugs, which are mainly used for the treatment of focal vascular anomalies [11], while liquid embolic agents include particulates, polymers or in situ gelling biomaterials, which are used for the treatment of numerous vascular disorders [12]. They can be temporary, as they promote rapid action and are used to occlude a haemorrhagic vessel, or they can be permanent, as they are used to treat complex vascular malformations [12]. Depending on the size and calibre of the target vessel, a careful selection of the embolising agent is made to ensure occlusion is limited to the desired site only. Specifically, embolisation of an actively bleeding site leads to an immediate mechanical effect, resulting in a decrease in arterial pressure upstream of the site and a decrease or cessation of blood loss [13]. This effect results in the formation of a haemostatic clot that promotes healing of the vessel wall [14]. Despite extensive applications in clinical trials, there are still some limitations inherent to the use of these embolising agents, such as the ease of aggregation, off-target embolisation, collateral circulation, migration and compaction of coils, the high incidence of bleeding or breakthrough haemorrhage and increased morbidity and mortality, especially in coagulopathic patients [15]. To overcome these limitations, hydrogels based on natural polymers such as alginate, chitosan and fibroin [16] have been developed as emerging and versatile embolic systems with favourable properties, including significant swelling capacity, permeability, mechanical strength and biocompatibility [17,18]. Hydrogels, consisting of three-dimensional network structures, can be delivered in a sol-fluid state using any clinically relevant catheter type and can be converted to a non-fluid gel state through in situ cross-linking [19] polymerisation and sol–gel transition [20].These hydrogels can prevent obstruction of the microcatheter and completely occlude the vessels through the formation of a solid mass at the treatment site, thereby reducing the risk of fragment migration and non-target embolisation [21]. The main purpose of this review was to focus on the advantages, disadvantages and safety profiles of conventional and novel embolic agents related to the application of these devices in the endovascular field. In addition, to expand on the clinical data reported in the literature, a series of cases, performed by the authors of this review concerning the correction and exclusion of type I and type II endoleaks after an endovascular procedure of abdominal aortic aneurysm exclusion (EVAR) with a coil (penumbra coil delivered by the PROGREAT microcatheter), renal artery malformation (AVM) exclusion with a coil (penumbra coil delivered by the ASAHI Master Parkway microcatheter) and endoleak correction by application of Onyx 18 in arteries where sealing by the endoprosthesis was not guaranteed.

## 2. Liquid Embolic Agent

Liquid embolic agents are classified into polymerising liquid embolisms and precipitating liquid embolisms [22]. The former consists of solutions of monomers or polymers that polymerise in the presence of an initiator in a covalently cross-linked solid material. They include various in situ gelling systems, which are thermo-responsive and undergo a phase transition from sol to gel temperature [23]. Precipitating liquid embolic, on the other hand, consists of polymeric solutions preformed in a solvent miscible with water. After injection into the human body, the solvent diffuses and the embolic material precipitates into a solid matrix [24]. Examples of liquid polymerising embolic agents are as follows: N-butyl-2-cyanoacrylate (NBCA), Fibrin Glue, N-isopropylacrylamide (NIPAAm) Copolymers, Poly(ethylene glycol)/Poly(propylene glycol)/Penta erythritoltetrakis (3-mercaptopropionate) (PPODA/PEGDA/QT), shear-thinning biomaterials, Silk Elastin Protein (SELP), and Poly(ethylene glycol) Hydrogel Embolisation System (HES), while Eudragit, calcium alginate, ethylene vinyl alcohol copolymers, Onyx poly(lactide-co-glycolide) and poly(hydroxyethyl methacrylate) andiodinated poly(vinyl alcohol) are considered liquid precipitating embolic agents [24].

The in situ formation of a gel allows the liquid formulation [25] to be easily administered through standard catheters or coaxial microcatheters, especially in the case of tortuous, distal and/or small-calibre vessels, which provided the liquid materials have a viscosity appropriate to the solution state and do not induce obstruction of the microcatheter through the aggregation of particles or microspheres [26]. The level of occlusion, proximal or distal to the tip of the release catheter can be chosen depending on the vascular pathology. Once the liquid undergoes a sol–gel transition, the resulting gelled mass can mould itself to fill the geometry of the vessel into which it is injected and any irregularities, thus adapting ideally to the contours of the vessel to be treated [27]. The mechanism of action deployed relates to a complete occlusion of the vessel that does not involve the formation of thrombi to complete the occlusion [28]. The formation of a single solid mass of embolic material at the treatment site reduces the risk of migration of fragments once implanted and offers advantages to all patients with coagulopathies or undergoing anti-coagulant therapy who show an impairment in the process of blood clot formation [28].

### Onyx^TM^ Gel

Onyx (Medtronic, Dublin, Ireland) is a commercially available liquid embolic product approved by the FDA in 2005, consisting of a (ethylene-co-vinyl alcohol) (EVOH) copolymer dissolved in DMSO with tantalum powder in suspension [29].

Prior to dispensing, Onyx must be agitated to obtain a uniform suspension of the polymer solution [30]. Its solidification occurs through an ‘outside-in’ precipitation process in which the DMSO rapidly diffuses into the blood and the EVOH precipitates and hardens on the outer surface [31]. Upon entry into the vascular system, the polymer precipitates from the solution because it is diluted by the aqueous conditions of the blood acting as a non-solvent for the polymer. In fact, chemically, the polar hydroxyl groups present in the vinyl alcohol unit are also responsible for the sensitivity of the polymer to moisture; therefore, water is easily absorbed due to the hydrophilic character of EVOH. With afurther increase in water concentration, EVOH will tend to plasticise in situ and inter- and intra-molecular bonds will weaken, thus causing a decrease in barrier properties.

**Figure 1:**
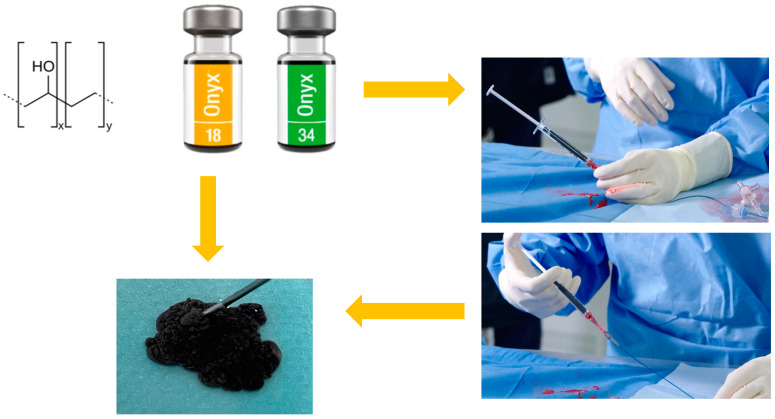
Graphical representation of the chemical formula, macroscopic appearance and material for the injection of Onyx^TM^gel.

The inner fluid breaks up the solidified outer surface upon further injection, and the outer surface solidifies again. With a ‘lava-like’ flow, Onyx can flow deeper into the wound [32]. Once fully solidified, Onyx appears as a sponge-like gel that traps the tantalum powder and solidifies completely within 5 min after injection. The tantalum present in the precipitated gel not only gives the material a long-term radiopacity so that embolised vessels can be easily located with X-rays, but also gives a black colouring to the suspension that could cause a tattoo-like colouration when treating sites close to the skin’s surface (Figure 1) [33].

The various commercially available formulations of Onyx allow the most appropriate viscosity to be chosen based on the size and speed of blood flow in the affected vasculature. The types widely used and marketed are as follows: Onyx-18, Onyx-20, Onyx-34 and Onyx HD-500, whose viscosities are 18, 20, 34 and 500 mPas. Specifically, Onyx 18 is composed of 6% ethylene vinyl alcohol (EVOH) and 94% DMSO; Onyx 20 is composed of 6.5% EVOH and 93.5% DMSO; Onyx 34 is composed of 8% EVOH and 92% DMSO; and OnyxHD 500 is a formulation composed of 20% EVOH and 80% DMSO, which is widely used for the embolisation [34]. Onyx 18 is a low-viscosity gel used mainly for treatments that require a high degree of distal penetration as in the case of AVMs; Onyx 34 reduces the risk of material migration and Onyx 500 is used for embolisation of wide-neck aneurysms or intracranial aneurysms. Although Onyx has a low inflammatory response, the doses of DMSO contained therein must be below the toxic level with an injection rate that must notexceed 0.3 mL/min to avoid vasospasms. In this regard, it is necessary to use catheterisation equipment certified and compatible with it [35].

The administration process can also be carried out with intermittent injections, which allow for a pause in administration (shaking for about 20 min) to assess the progress of the embolus and then continue with further penetration of the embolus into the vasculature. When the solution is injected through the catheter, a small mass of polymeric gel forms that adheres to the tip and swells when more solution is administered. It solidifies completely within 5 min after injection and forms a spongy gel that traps tantalum powder [36]. A film forms at the solution–blood interface as the material precipitates, forming a bag full of solution that is further expanded by the growing mass of fresh solution exiting the catheter tip [37].

This causes the forming epidermis to continuously rupture as the embolus continues to expand, with subsequent reformation of the precipitating solution.

Once a sufficient amount of material has been delivered, the endpoint of embolisation is determined when the delivered embolic fluid no longer flows within the vessel due to solidification and is easily visualised in real-time fluoroscopy with X-ray imaging techniques [38]. When it comes to the limitations related to the use of Onyx, it is important to point out that in addition to the cytotoxicity exerted by the presence of DMSO, this agent could be disadvantageous since it could cause proximal venous occlusions with increased recruitment of collateral vessels, retrograde thrombosis, embolic stroke, etc. In addition, it could cause a breakthrough of normal perfusion pressure, theembolisation of normal vessels causing intimal damage with thrombosis, could pass into the venous circulation causing pulmonary emboli and also could cause femoral sheath-related morbidity (e.g., infection, femoral or retroperitoneal haematoma) [29].

## 3. Coil Embolic Agent

Coils are considered to be embolisation agents that are easy to use, visible through fluoroscopy and immediately available. The coils are made of steel or platinum and offer permanent occlusion once deployed [39]. They are available in a wide range of shapes, sizes, coating materials and deployment methods used for effective embolisation in various vascular pathologies. Coils offer permeant embolisation through three synergetic mechanisms: (i) slowing down the flow through the vessel due to the mechanical blockage, (ii) acting as a thrombogenic scaffold for clot formation for clot formation, and (iii) inducing damage to the vessel wall that causes the release of thrombogenic factors [40]. The thrombosis occurs within 5 min of deployment, although this timeframe may vary depending onthe type of coil used, the size of the embolised vessel and the degree of flow through the embolised structure [41]. The coils are generally made of platinum or stainless steel; the former, while more expensive than stainless steel, is softer and more durable, and offers better visualisation in fluoroscopy. Furthermore, since coil embolisation depends on the patient’s ability to form a thrombus, coagulopathic states such as thrombocytopenia, platelet dysfunction and coagulation factor abnormalities can impede complete vessel occlusion [42].

**Figure 2:**
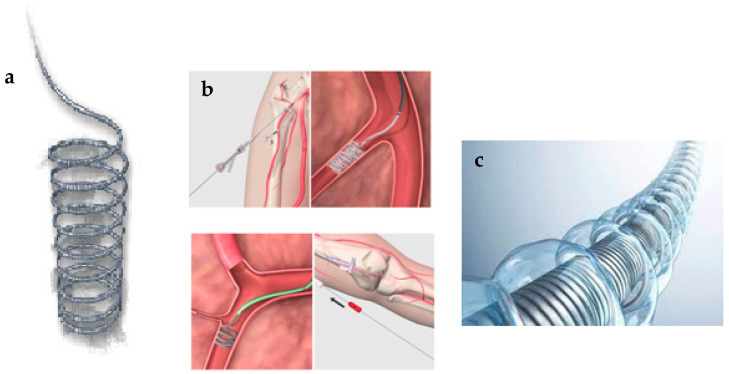
Coil bare (**a**), coil endovascular application (**b**), hydrogel-coated coil (**c**) for embolisation.

Coils can be bare or coated with various synthetic and non-synthetic polymeric materials that increase their thrombogenicity (Figure 2). In addition to Dacron, nylon, polyester, polytetrafluoroethylene, etc., coils can also be coated with hydrogels, which allow them to expand up to four times their size, unaffected by innate thrombolytic processes that can lead to recanalisation of the embolised segment. Coils are classified into different levels of stiffness, including ultrasoft, soft, standard, firm and stretch resistant [43]. There are different types of coil: (i) pushable, (ii) injectable, (iii) detachable, (iv) liquid and (v) hydrogel. Pushable coils consist of a guide wire that mechanically pushes the coil into the desired positionand are most commonly used in interventional radiology; injectable coils can be injected through a catheter using a small syringe filled with saline, saving time due to the absence of a booster cable; detachable coils are coils attached to an introducer wire that are deployed with a specific release mechanism at the operator’s discretion; and liquid coils are also deployed by forced injection of contrast through the catheter after loading the coil [44]. Coils are typically used to occlude larger vessels and cause complete occlusion of the vessel, equivalent to surgical ligation, inducing thrombosis [45]. In the literature, hydrogel coils are useful as a replacement or platinum coils in the treatment of aneurism [46]. Limitations related to the use of coils, whether bare or coated are most often related to cost, inability to be retrieved without removing the entire catheter, the possibility of creating local artifacts on MRI, the inability to be repositioned especially in tortuous vessels with sharp curves and the possibility of being prone to reflux and retraction of the catheter upon deployment. In addition, they may have more complicated mechanisms for deployment and are highly dependent on the operator’s experience, and they may fail in cases of severe patient coagulopathy. As for those covered with hydrogels, possible limitations could be related to the time of coil preparation, which takes place in warm saline before deployment (1–5 min), and the time of complete volumetric expansion of the hydrogel, which takes about 20 min after installation to swell [39].

## 4. Hydrogel Embolic Agents

The new liquid embolic agents used in the last decade are represented by hydrophilic gels called hydrogels, with a three-dimensional network structure capable of rapidly expanding and re-swelling in water, retaining a large volume of it. Furthermore, they have good permeability, good mechanical resistance and high biocompatibility with biological tissues [47]. By altering their physicochemical structural properties and introducing functional components into the polymer matrix, it is possible to convert them into hydrophobic gels. Specifically, recent studies have indicated that the introduction of different physical interactions, such as hydrophobic interactions, hydrogen bonds and dipole–dipole interactions, into hydrophilic hydrogel networks can give rise to stimuli-responsive shape memory hydrogels [48]. In this regard, they can undergo a gel–sol phase transition if influenced and/or subjected to stimuli such as temperature, pH, ionic forces, light irradiation, mechanical stresses and magnetic field, which allows these hydrogels to gel in situ and to fill the vascular system with various geometries and dimensions, completely occluding the vessels. Hydrogels, as embolic agents, can be designed to load therapeutic drugs, bioactive molecules and functional nanomaterials within them for controlled delivery to the release site or local procoagulant activity [49]. Depending on the materials used in hydrogel preparation, there are two types: natural hydrogels and synthetic hydrogels. Natural hydrogels, derived from substances such as alginate, hyaluronic acid (HA), agarose, chitosan, collagen, fibrin and polyethylene glycol, show greater biocompatibility than synthetic hydrogels, as they mimic the ECM of natural tissues and influence the cell adhesion, proliferation and differentiation [50]. Synthetic hydrogels are made up of synthetic hydrophilic macromolecules that undergo physical or chemical cross-linking. These macromolecules include, among others, poly(acrylic acid) (poly-AA) and its derivatives, poly(vinyl alcohol) (PVA), polyoxymethylene and polyacrylamide. Their higher water absorption capacity, longer shelf life and wider range of chemical raw materials make them widely used [51]. Numerous examples of hydrogels as embolic agents are reported in the literature. For example, Oklu and co-workers have fabricated physically cross-linked hydrogels combined with nanosheets or nanoclay particles that can effectively embolise the femoral arteries of mice and the renal arteries of Porcines [52,53]. Duan et al. prepared a temperature-sensitive injectable hydrogel composed of poloxamer 407 (F127), hydroxymethylcellulose (HPMC) and sodium alginate (SA) that successfully blocked rabbit hepatic and renal tumour arteries [54]. Although hydrogels are state-of-the-art embolic agents, it is necessary to take into consideration certain parameters that can make them more suitable for clinical applications. Specifically, it is necessary to make them highly biocompatible with native human tissues so that they do not induce toxicity in the surrounding tissue when implanted in human blood vessels; it is also necessary to prevent their degradation products from causing adverse effects on human health [1]. When it comes to the character of hydrogels in blood vessels, it is important to take into consideration the rheological properties of the same, emphasising how they under a particular applied pressure change their shape to enter and pass through a tubular environment (e.g., capillary) with a diameter smaller than the size of an unperturbed object such as blood vessels. The latter would exhibit frequency-dependent viscoelastic properties such as in the case of human arteries, the dynamic elastic modulus increases in a low-frequency range (<3 Hz) and then remains almost constant up to 10 Hz. Rheological evaluation of the hydrogels before and after secondary cross-linking would provide more information on the mechanical properties of the produced hydrogels, shear stresses and viscoelastic properties of the hydrogels. From rheology, we know that a 7.5 wt% hydrogel has a higher viscosity and a higher modulus of accumulation and loss than a 5 wt% hydrogel. For 7.5 wt% hydrogels, increasing the flow rate from 2 to 8 mL/h increased the injection force. Other factors affecting injection force included needle calibre and length. Increasing the needle gauge, and thus reducing the needle diameter, increased the injection force [55]. In this review, we will focus more on natural hydrogels.

### 4.1. Chitosan Hydrogel

**Figure 3:**
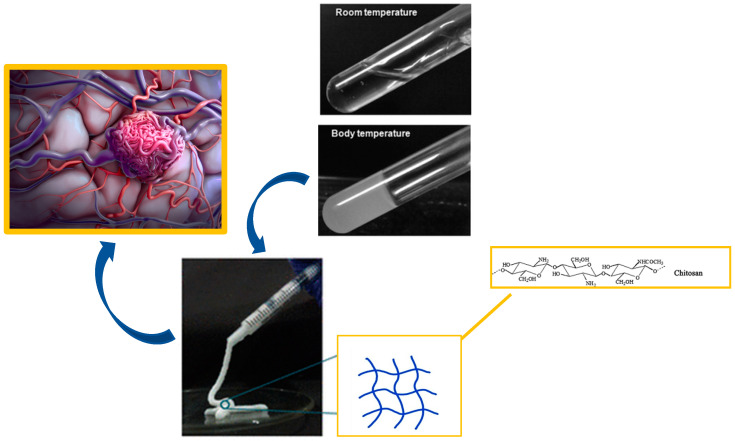
Chitosan hydrogel as embolic agent for embolisation process.

Chitosan is considered a natural heteropolymer resulting from the alkane deacetylation of chitin consisting of a chain with D-glucosamine and N-acetyl-D-glucosamine residues [56]. It has numerous advantages as an embolisation agent, as it is able to dissolve in acidic solutions as a polyelectrolyte and undergo neutralisation of the amine groups at pH = 6.2, thus removing electrostatic repulsive interchain forces [56]. This allows extensive hydrogen bonding and hydrophobic interactions between the chains to produce a hydrated gel-like precipitate [57]. When combined with a weak base such as the β-glycerophosphate salt (*β*-GP), it forms a system (CS/*β*-GP) that remains in solution at room temperature and physiological pH, but turns into a gel upon heating to physiological temperature, undergoing heat-induced gelling. The temperature-induced phase transition behaviour of the CS/*β*-GP system can be attributed to multiple interactions, including electrostatic interactions between the ammonium groups of the chitosan and the phosphate groups of the *β*-GP, enhanced hydrophobic interactions of the CS-CS, and increased intra-molecular hydrogen bonds in the CS [58]. The temperature-responsive sol–gel transition properties of CS/*β*-GP make it a potential embolic candidate (Figure 3).

For in vivoembolisation procedures, gelling is an interesting property, as it allows low viscosity during the injection but limits gel migration and associated embolisation risks. Secondly, chitosan is biocompatible and forms a porous matrix that can be infiltrated by cells. In addition, it is haemostatic and may promote thrombosis in the aneurysmal sac. Its mucoadhesive properties should allow for good flow occlusion and limit the migration of hydrogel by binding to surrounding tissues. Finally, it is a relatively inexpensive product that can be heatsterilised. However, chitosan hydrogels are neither radiopaque nor sclerosing, so they should be combined with agents having these properties [59,60,61].

The addition of commercial contrast agents (e.g., Isovue^TM^, Visipaque^TM^ and Conray^TM^, tetradecyl sulphate) to chitosan/*β*-GP significantly increases the viscosity of the solution prior to gelation, reducing the gelation rate and helping to visualise the embolisation procedure. Furthermore, they are rapidly released from the hydrogel and do not hinder further imaging or endoleak detection. In a study by Fatimi et al., a CS/*β*-GP hydrogel containing Iopamidol as a radiopaque agent and sodium tetradecyl sulphate as a sclerosing agent was prepared. The results showed that the hydrogel gelled rapidly at 37 °C and that the mechanical and sclerosing properties were excellent. Embolisationwas performed on a bilateral canine aneurysm modelwhere it was observed that the hydrogel showed good visibility during injection and no endoleak at three months after control, while type I endoleaks were observed in two in aneurysms treated with hydrogels without tetradecyl sulphate. These results verify that the developed versatile hydrogels possess promising potential for the treatment of endovascular aneurysms [62]. In another study conducted by Ai et al., an injectable dual-network composite (CDN) hydrogel was prepared as a liquid embolic agent by cross-linking of poly(vinyl alcohol) (PVA) and carboxymethylchitosan (CMC) by dynamic covalent bonding of borate ester and benzoic-imine. A two-dimensional nanosheet, i.e., a layered double hydroxide (LDH), was incorporated into the network through physical interactions that led to a strong reduction in yield stress for hydrogel injection and the ability to load therapeutic agents such as indocyanine green and doxorubicin (DOX) for photothermal therapy (PTT) and chemotherapy functions. The results showed that the hydrogel could be transported through a thin catheter and strengthened in situ under physiological conditions, such as in blood by secondary cross-linking with phosphate ions for longer degradation time and better mechanical properties. Tests in a rabbit model revealed the embolic capacity of the hydrogel by showing an injection and efficient embolisation of the rabbit renal artery, which could be traced to X-ray fluoroscopy. Furthermore, the presence of doxorubicin in the hydrogel is able to effectively inhibit subcutaneous tumour growth through the combination of photothermal therapy and chemotherapy in animal models [63]. In the study conducted by Ning et al., chitosan/βglycerophosphate (C/GP) was modified by tissue engineering to introduce a ‘live cell’ component into it. Subsequently, tests were carried out on the malformed vascular lumen of pig subjects, using a microcatheter technique that enabled it to regenerate in an ‘autologous living tissue’ with a predetermined mechanical strength, thus permanently occluding the deformed blood vessels. The preliminary data of this study supportthe hypothesis that the application of C/GP and fibroblasts can be effective in the treatment of cAVMcerebral arteriovenous malformation using the rete mirabile (REM) as a model [64].

### 4.2. Alginate Hydrogel

Alginate is a natural anionic copolymer derived from brown algae, composed of α-L-Gulonuronic acid (G-units) and β-D-Mannuronic acid (M-units), irregularly linked by β-1,4-glycosidic bonds in various arrangements along the polymer chain. The length of the bound G-units correlates directly with mechanical properties, while the M-unit content correlates with immunogenicity [65]. The main alginate chain contains a large number of hydrophilic groups, such as hydroxyl and carboxyl groups, which are easy to modify through ionic reticolations with divalent cations such as calcium ions, barium ions, strontium ions, etc., or through metal co-ordination. Although this cross-linking strategy is low-cost and simple in the process, the hydrogel obtained usually has low mechanical properties and thermal stability and is prone to degradation under physiological conditions [66]. Therefore, many researchers have gradually turned their attention to other cross-linking strategies, such as chemical cross-linking and interpenetrating network (IPN) technology.

For example, calcium alginate-based hydrogels exhibit excellent embolisation properties, biodegradability and rapid occlusive gel formation without catheter entrapment [67,68]. The alginate-based embolic agents can also cut off the blood supply of the tumour tissue and release the drug gradually at the embolisation site, which isdeveloped as a dual-effect drug for tumourembolisation and chemotherapy [69]. Injectable hydrogels based on cationic polymers are favourable for loading functional agents, which is a convenient and effective approach to endow them with versatile properties. In a study conducted by Fan et al., a hydrogel was made from a liquid metal introduced into a calcium alginate (CA) solution using CaCl_2_ for cross-linking. The presence of the liquid metal is very important because it gives the hydrogel a high X-ray radiopaque property, which ensures its real-time tracking in the blood vessels during the embolisation procedure. The hydrogel was tested on rabbit ears and showed significant inhibition of growth auricular tumour growth after embolisation with the disappearance of the same after a 15-day treatment [70]. An injectable photochemical alginate-based hydrogel implant with a long lifespan was developed for blood vessel embolisation and intraoperative real-time imaging. The researchers first prepared a type of photochemical hydrophilic nanoparticles (PA-NPs) composed of PdPc(OBu)8 as a photosensitiser, PCU as a photoenergy cache unit and Eu(TPPO)2(β-NTA)3 as a photoemitter. Subsequently, the PA-NPs were dispersed in an alginate solution, which could be primed by Ca^2+^ in the blood to form a strong hydrogel (PA-gel). This hydrogel was able to form in situ in the abdominal aorta of mice to embolise the blood vessels. The localisation of the vascular embolisation was observed by the increased afterglow signal at the implant. The implantation and long-lasting afterglow allowed real-time monitoring of dynamic blood flow information. The excellentbiocompatibility and imaging capability of this alginate-based hydrogel can be used as a potential embolic agent in clinical medicine [71]. Alginate-based hydrogels can undergo two sequential cross-linking processes: the initial reversible cross-linking process to achieve the shear-thinning property and thus injectability, and the in situ irreversible covalent cross-linking to achieve desirable mechanical properties. In work conducted by Xie et al., an injectable alginate-sulphate-based hydrogel undergoing insitu covalent cross-linking was designed, resulting in the formation of a double-cross-linking (DCN) hydrogel. The latter showed efficient haemostatic capacity and good biocompatibility. In addition, tests showed it to be radiopaque and thus capable of aiding X-ray imaging during endovascular embolisation processes. The latter was finally evaluated in vivo in rabbit arteries, where the DCN hydrogel effectively embolised the arteries and induced fibrosis and tissue regeneration for permanent embolisation [72].

### 4.3. Silk-Elastin-like Protein Hydrogel

Silk-elastin-like proteins (SELPs) consist of alternating motifs of silk and elastin isolated from silkworms (GAGAGS) and mammalian elastin (GVGVP). The first component provides temperature-activated physical cross-linking sites, while the second provides dynamic functions [73]. The biological and physicochemical properties can be adjusted by varying the silk/elastin ratio, modifying the second residue in the elastin sequence, changing the molecular weight and adding peptides to expand functions [74]. SELPs undergo a non-reversible transition from liquid to robust gel, mediated by hydrogen bonding between silk motifs, resulting in the formation of antiparallel β-sheets. This passive sol-to-gel transition of SELPs was used to occlude physiological flow through selected microfluidic channels after injection and warming to body temperature [75,76]. Silk elastin-like protein polymers (SELPs) combine the solubility of elastin and the strength of silk to create an easily injectable liquid embolic that transitions into a solid depot suitable for loading with drugs, gene therapy agents or pro-biological ducts. SELP, injected as a liquid solution, penetrates the vascular system before transitioning to a solid hydrogel. In a work conducted by Hatlevik et al., the embolisation, stability and biocompatibility of a SELP-based hydrogel were evaluated at survival intervals of 7, 30 and 90 days in a porcine model. SELP embolisms selectively block blood flow to the kidneys and liver, without off-target infarctions. As assessed by angiography, SELP renal embolisation shows decreasing persistence over the duration of the 90-day study period [77]. According to Jensen et al., silk elastin-like protein-based hydrogel (SELP 815K) was used to embolise aneurysms in a rabbit elastase model. SELP 815K effectively embolised the aneurysms of the in vivo model, achieving >90% occlusion, using commercial microcatheters. No device-associated adverse effects were observed in any of the animals, and SELP 815K showed no cytotoxicity. SELP embolisation showed no deleterious effect on local tissues and features consistent with reendothelialisation of the aneurysm neck were noted in histological examination one month after embolisation. SELP 815K shows promise as an embolic treatment for the treatment of unruptured cerebral aneurysms [78].

### 4.4. Polaxamer 407-Based Hydrogel

Poloxamer is a linear triblock copolymer composed of a block of polypropylene oxide (PPO) between two blocks of poly(ethylene oxide) (PEO). When its concentration is higher than the critical micelle concentration (CMC), the poloxamer can self-assemble to form micelles due to the hydrophobic nature of the PPO block. The micelles join together, eventually forming a gel if the concentration is sufficient [79]. This gelation can be induced by temperature because the CMC of the poloxamer decreases with increasing temperature [80]. The injection of Poloxamer 407 gel causes a complete occlusion but the interaction with water leads to the disintegration of the gel structure and complete recanalisation within 10–90 min [81]. For this reason, it is mainly used as a temporary embolic agent in the treatment of intracranial aneurysms and tumours of the carotid body and/or in restoring the patency of the occluded walls of the vessels [82]. Temporary embolic agents based on polaxamer 407 also possess favourable properties as they are non-thrombogenic, atraumatic and capable of inducing a reliable reversible occlusion. Wang et al. have created a composite hydrogel (FHSgel) based on P407 F127/hydroxymethylcellulose/(HPMC)/sodium alginate (SA), which is sensitive to temperature and exhibited sol–gel transition behavior with increasing temperature. The presence of HPMC allows the gelation temperature (Tgel) to be effectively regulated, while SA improves gel strength. The tests carried out have shown that FHSgel is able to penetrate through the canine hepatic arterial branches and block reducing blood flow and the risk of ectopic embolism when diluted in the blood circulation [83].

## 5. Endovascular Application of Embolic Agents

### 5.1. Aneurysm

An aneurysm is an irreversible and permanent process in an artery that leads to a dilation of 1.5 times the vessel’s native calibre, resulting in rupture and internal bleeding. Aneurysms can occur throughout the body but are most common in the brain, aorta, renal artery, legs, spleen and brain. The abdominal aorta is defined as ‘aneurysmal’ if its calibre is greater than 3 cm [84,85]. The section of the aorta most affected by aneurysm formation is the infrarenal section, which is approximately 1–2 cm from the emergence of the renal arteries. This dilatation mainly recognises three pathophysiological drivers: proteolysis, inflammation and smooth muscle cell (SMC) apoptosis, and is completely silent and asymptomatic until the aneurysm itself ruptures, leading to death in 65% of patients [86]. The goal of treating aneurysms is their complete and permanent occlusion with an embolic agent. Typically, detachable metal coils are used that can be introduced with a pusher to fill the aneurysm sac, resulting in reduced blood flow within the aneurysm. To increase the density of the aneurysm filling, the bare coils can be coated with cross-linked hydrogels whose volume can be expanded without dissolving upon contact with the fluid. Improving the volumetric filling of the aneurysm sac should allow a higher filling density, reduce the rate of aneurysm recurrence and prevent vessel rupture [87]. The principle of aneurysm embolisation is to occlude both the afferent and efferent vessels of the aneurysm so that the aneurysm is completely excluded from blood circulation. However, these coils often have the disadvantage of easily migrating through the bloodstream and causing pulmonary embolism, stroke or myocardial infarction. The most common complications after endovascular aneurysm repair are type I and type II endoleaks [88]. Type I endoleaks result from an inadequate seal and may be found at the proximal end of the graft (type Ia) or the distal end of the graft (type Ib). In EL1 endoleaks, the resulting communication between the high-pressure aortic lumen and the perigraft space may result in increasedpressurisation and expansion of the sac [89]. They require immediate treatment to avoid the risk of sac rupture. Endoleak after EVAR may occur in about 20 percent of patients, with type II endoleak(ELII) comprising upto 40 percent of these endoleaks. ELII is described as retrograde collateral flow from aortic branches and most commonly originates from the lumbar arteries or the inferior mesenteric artery [90]. Transcatheter embolisation is considered the primary treatment for type II endoleaks, while for type I endoleaks, an appropriate treatment option is a transarterialembolisation [91]. A recent study by Aydin et al. described using a self-expandable stent to cover the neck of the aneurysm and induce thrombosis while maintaining sufficient blood flow in the main vessel. The results obtained showed that occlusion occurred in all patients treated despite the risks arising from the unpredictability of the aneurysm occlusion process [92]. To limit the risks associated with the use of coils and promote complete filling of the aneurysm, the use of liquid embolic agents such as Onyx has been recommended in recent years. In a study by Mozes et al., the use of Onyx was demonstrated to be effective in the treatment of a type IIendoleak, inducing stabilisation and a reduction in the diameter of the aneurysm sac in 66% of patients and up to 72% in isolated type IIendoleaks [93]. In the study conducted by Scallan et al., the results obtained were compared after using Onyx and spiral for the treatmentof type II endoleaks associated with aortic sac growth.The researchers observed that the action of Onyx was superior to coil embolisation, with lower rates of treatment failure, defined as aneurysm sac expansion > 10 mm. The request for further reoperations was significantly greater in the group where the embolisation was performed with the coil; while no differences in AAA-related death or secondary rupture were found between the two treatment methods [94]. However, some problems, linked to the potentially toxic effects of the solvent used to prepare Onyx, may limit its use, and therefore require the design of agents that are more biocompatible with organic fluids. For this reason, studies have also been conducted on the use of injectable alginate-based hydrogels in the treatment of aneurysms. In this regard, Barnett and Gailloud developed an embolic system by coupling a commercial alginate-based gel with a solution containing the enzyme alginate lyase and ethylenediaminetetraacetic acid. This gel was able to selectively dissolve migrating into normal artery moles, as confirmed in a rabbit aneurysm model, which proved to be practical and safe [95]. In another study conducted by Sivakumaran et al., the mechanical properties of the constituents of aneurysmal sacs after endoleakembolisation, with chitosan (CH)-based hydrogel and chitosan hydrogen-based hydrogel with the sclerosant sodium tetradecyl sulphate (CH-STS), were evaluated. The results obtained by in vitro rheometry showed a significant deformation of the CH-STS compared to the CH, as the presence of STS would improve the mechanical properties of the CHs by increasing the aggregation of the chitosan chain. Therefore, embolisation with CH-STS produced fewer residual endoleaks, highlighting better mechanical properties conferred by CH-STS on the contents of the aneurysmal sac compared to embolisation with a similar non-sclerosing agent [96].

### 5.2. Arteriovenous Malformation

Arteriovenous malformations (AVMs) are abnormal shunts between arteries and veins that bypass the capillary bed and affect the head, neck and intrathoracic area [97]. Because of their high flow, these vascular malformations cause significant rerouting or shunting of blood flow away from surrounding tissue. The main types of high-flow vascular malformations are arteriovenous malformations (AVMs) and arteriovenous fistulas (AVFs) [98]. AVMs consist of abnormal arteries and veins, with blood shunting occurring through a central collection of dysmorphic vessels (i.e., the nidus). In an AVF, a shunt of blood occurs through a single arterialised vein, rather than into a nidus, and is most commonly seen in the central nervous system [99]. The absence of capillaries causes high-pressure blood flow in the veins, causing them to widen and often leadingto rupture of the vessel [100]. Current treatment options focus primarily on catheter-guided surgery or stereotactic radiosurgery aimed at resecting, embolising, or irradiating AVMs to manage the risks associated with them [101]. Furthermore, in some cases, in surgical resection, the margins are generally difficult to determine and are therefore ineffective. However, reperfusion due to recanalisation or feeding from adjacent arteries leads to the recurrence of AVMs in approximately 25% of patients within the first year post-surgery. The goal of endovascular embolisation of AVMs is the closure of the entire nidus without occlusion of other surrounding normal and important vessels. Furthermore, endovascular management facilitates the localisation of the lesion and allows access to deep and/or critical areas. Coil embolisation has been widely used in AVMs achieving a technical success rate of 99%. Unfortunately, however, complications associated with coil embolisation include coil recanalisation, vascular damage and coil migration [102]. To overcome this problem, it is possible to use hydrogel-coated coils capable of increasing the packing density in the vascular lumen, ensuring better results and reducing the speed of recanalisation compared to bare coils. In a retrospective study conducted by Shimohiraet al., 57 patients with pulmonary AVM underwent embolisation using hydrogel-coated coils (AZUR^®^). The results obtained showed that no recanalisation occurred during an average follow-up period of 19 (range, 2–47) months. Furthermore, this study highlighted how the expanded hydrogel polymer contributes to the increase in the volume of the coil resulting in tight embolisation of the lesion [103]. Even the study conducted by Iguchi et al. confirms complete embolisation of the PAVM in 19 patients subjected to coated coil treatment without recanalisation or reperfusion processes having occurred in any of the 19 PAVMs 1 year after the control follow-up using pulmonary arteriography [104]. However, hydrogel-coated coils have disadvantages, such as slight resistance when dispensing the coil and time limitations; in fact, an injury to the venous sac is possible due to the rigidity of these coils, while a repositioning time of ≤3 min is necessary so that they can expand completely upon contact with the blood (within 20 min). For this reason, a growing body of evidence demonstrates that Onyx could be the embolic material suitable for the treatment of AVMs, as it is administered in a controlled and precise manner directly into the malformation. In a study conducted by Velinov et al., a clinical case of DAVF of the transverse sinus with ipsilateral ocular symptoms was considered where ONYX was used to induce a fistula occlusion rate of 55–85% with preservation of the transverse sinus [105]. In the clinical case analysed by Rajab et al., a combined transfemoral and transradial approach using Onyx liquid material has been shown to be an effective and safe method for the treatment of anterior chest wall AVMs. In fact, the use of Onyx improved distal embolisation within the AVM nest, while the use of a coil was necessary to decelerate the arterial flow and have complete management of the AVM [106]. Even with regard to the treatment of AVMs, there are limitations related to both the embolisation agents currently available and the devices used to administer them. For this reason, new studies are reported in the literature regarding the design and implementation of new embolisation agents in the form of hydrogels. In this regard, Ku et al. developed a hydrogel delivery method for intravascular microcatheters with dynamic modulation of the physical characteristics of the hydrogel at the catheter tip by photocross-linking with an integrated UV-emitting optical fibre. This allowed rapid transition from liquid to solid state to block blood flow to the vascular target, as well as dynamic modulation. Tests performed on animal models with neurovascular AVMs produced satisfactory results showing through post-procedural angiography significant occlusion of target vessels without evidence of complications, allowing the hydrogel precursor to effectively deposit at the level of the AVM in the animal. Furthermore, this study established the formation of a hydrogel with a viscosity range of up to 10^4^ Pa·s following dynamic modulation of photocross-linking [107].

## 6. Conclusions

This review article aimed to analyse and discuss embolic agents used for embolisation in the endovascular field. Specifically, different types of embolising agents were taken into consideration, including coils, Onyx and hydrogel, highlighting their advantages and disadvantages from a chemical–physical, clinical, toxicological and safety point of view. Although from a commercial point of view coil and Onyx remain the main agents used in clinical practice, it has been observed that in some circumstances they do not guarantee adequate safety and feasibility profiles to maintain human health unaltered. In fact, side effects often occur related to the ease of aggregation, off-target embolisation, collateral circulation, migration and compaction of the spirals, or the presence of solvents such as DMSO (in the case of Onyx), which cause a significant response inflammatory in patients. For this reason, in recent years, scientific research has led to the development of new biomaterials capable of satisfying the growing clinical demands of minimally invasive endovascular embolisation. A variety of embolic materials have been gradually developed, and more research interests have focused on the development of intelligent and/or multifunctional materials. An example is given by embolic agents based on hydrogels, biomaterials of natural or synthetic origin, which are easily customizable with favourable properties and wide versatility. They should have adequate gelation triggers to avoid catheter blockage and adequate sensitivity to occlude the target vasculature with various geometries and sizes. Furthermore, being biocompatible with native human tissue, they would not cause toxicity when implanted in human blood vessels and their degradation products would not cause harmful effects on human health. Unfortunately from a commercial point of view, it is not yet possible to find them on the market because they are in the development and testing phase. For this reason, common embolisingagents continue to be used in endovascular clinical practice, which, despite the disadvantages described in this review, continue to represent a valid option for the treatment of vascular pathologies that are at high risk for the patient, as can be seen from the clinical cases of embolisation recommendations in Appendix A and performed at the Annunziata Hospital of Cosenza and the Magna Graecia University of Catanzaro by a multidisciplinary team.

## Figures and Tables

**Figure A1:**
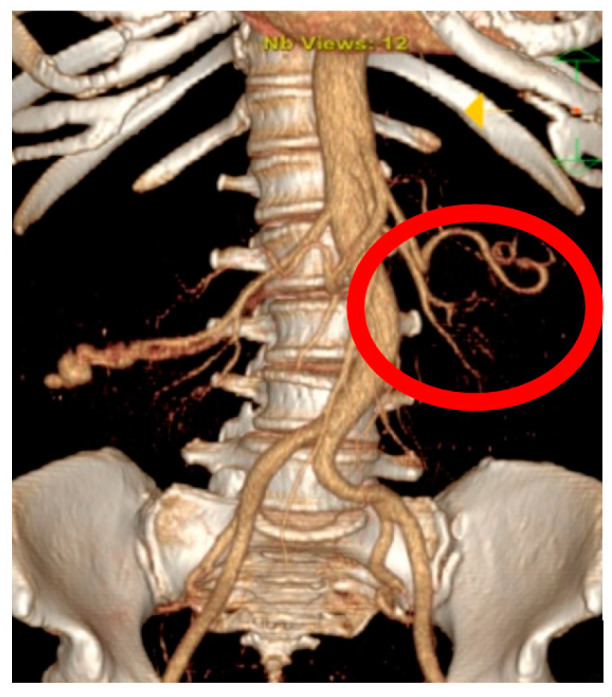
Pre-operative 3DMPR CT reconstruction.

**Figure A2:**
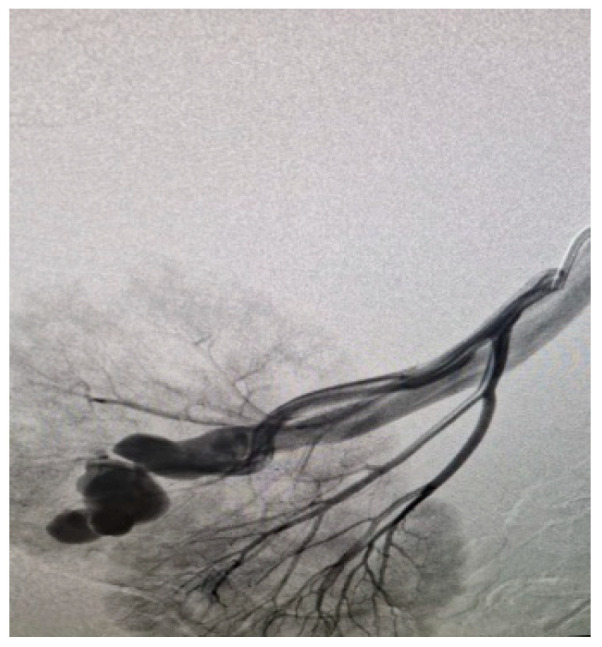
Intraoperative diagnostic angiography.

**Figure A3:**
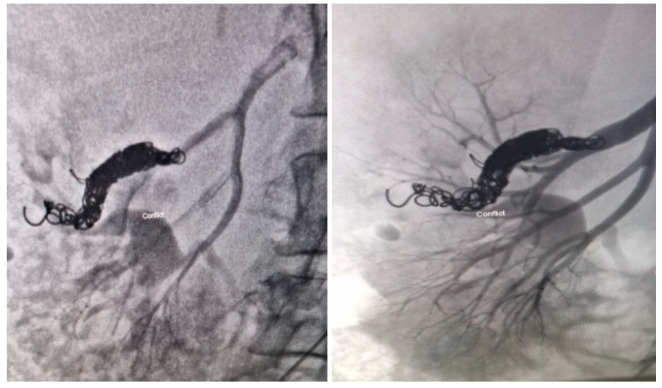
Angiographic control post-coil release.

**Figure A4:**
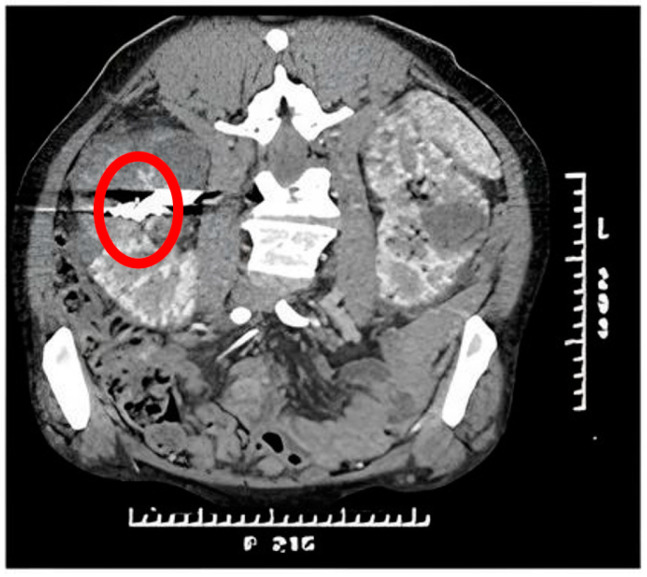
Control CT scan with contrast medium at 30 days with evidence of coils on release and absence of AVM.

**Figure A5:**
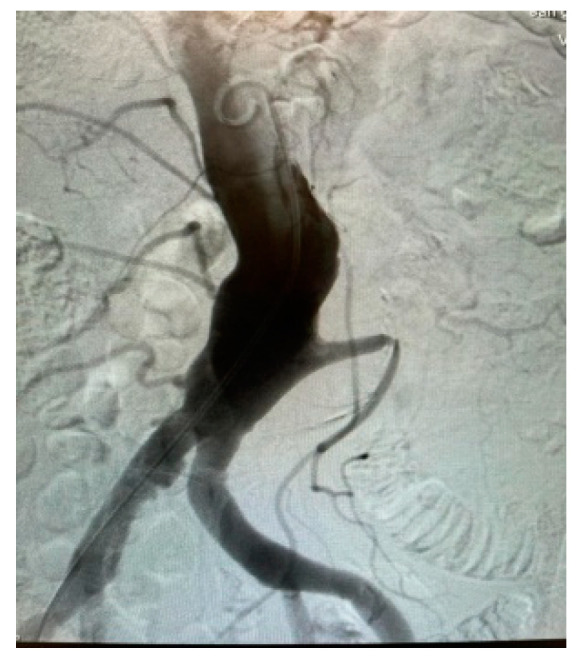
Intraoperative angiography via Pigtail 5F catheter.

**Figure A6:**
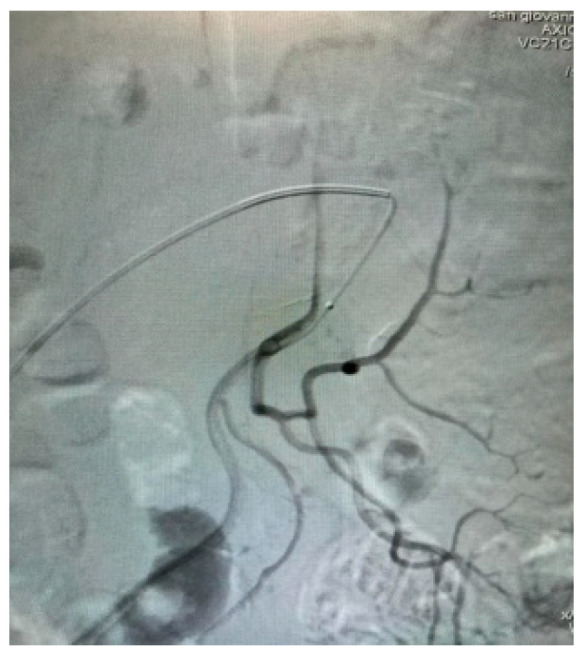
Selective AMI angiography with microcatheter and coil delivery.

**Figure A7:**
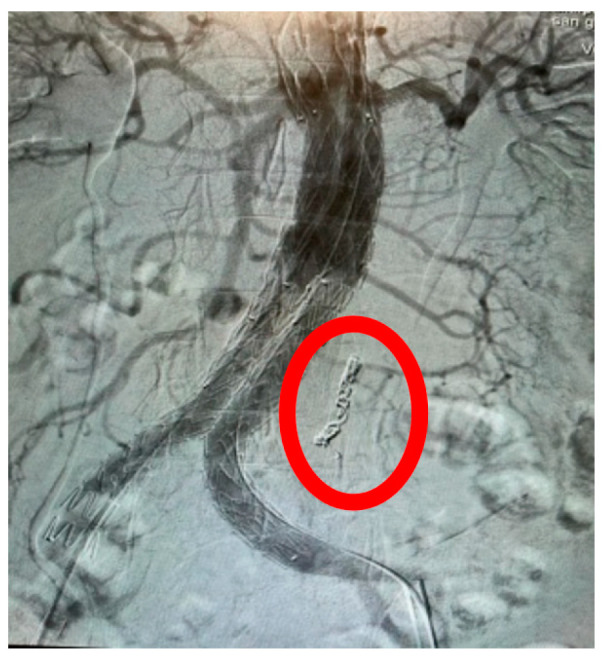
Post-operative angiography with exclusion of AMIvisualisation of coils and exclusion of AAA with endoprosthesis. Absence of endoleak.

**Figure A8:**
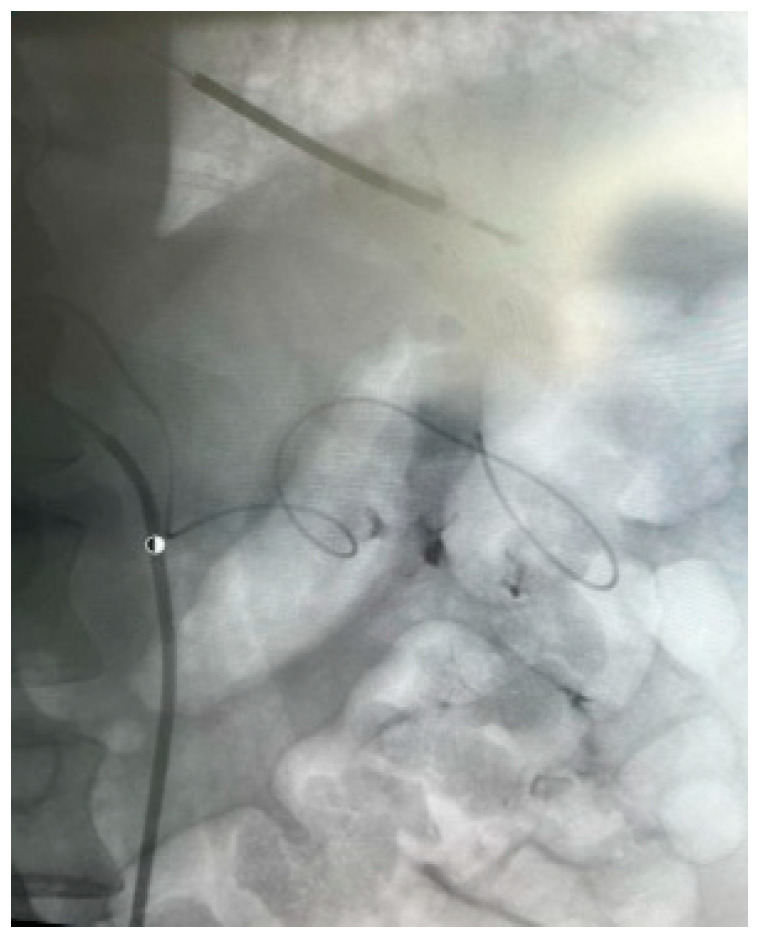
Selective catheterisation of the splenic artery.

**Figure A9:**
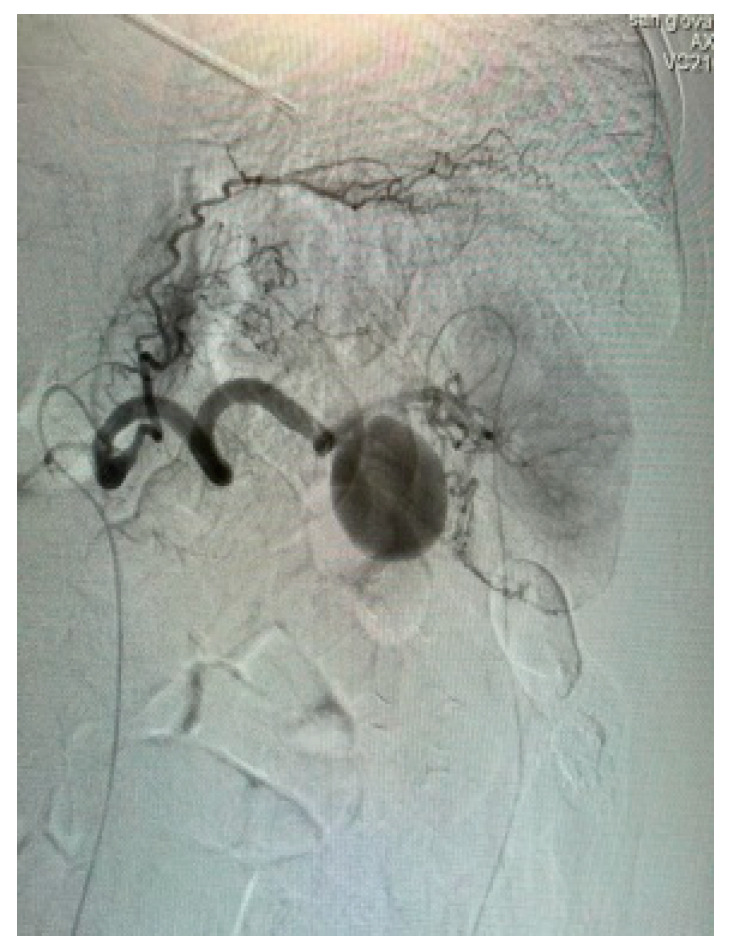
Intraoperative angiography with evidence of aneurysm.

**Figure A10:**
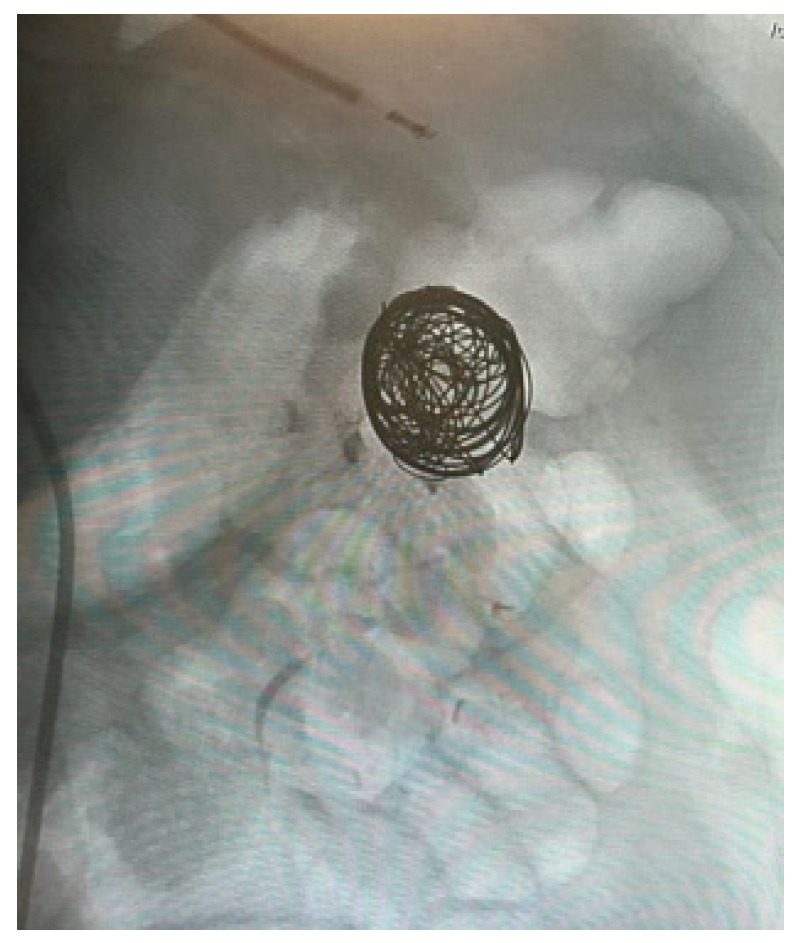
Detachable Coil EV3 release.

**Figure A11:**
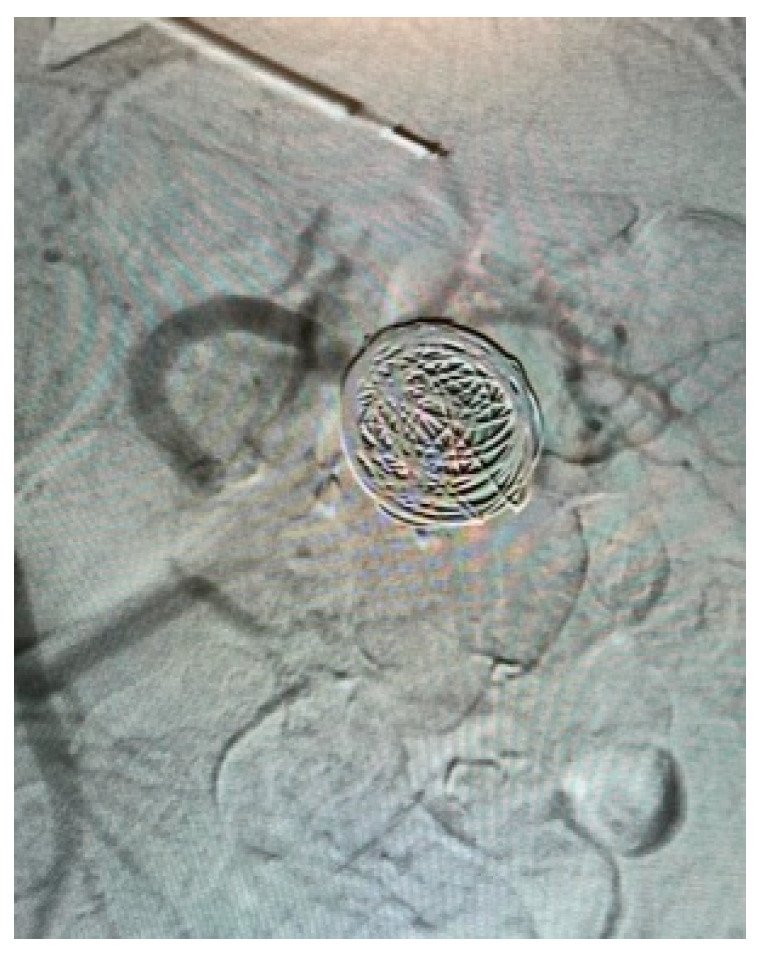
Post-embolisation angiography with exclusion of visceral aneurysm.

**Figure A12:**
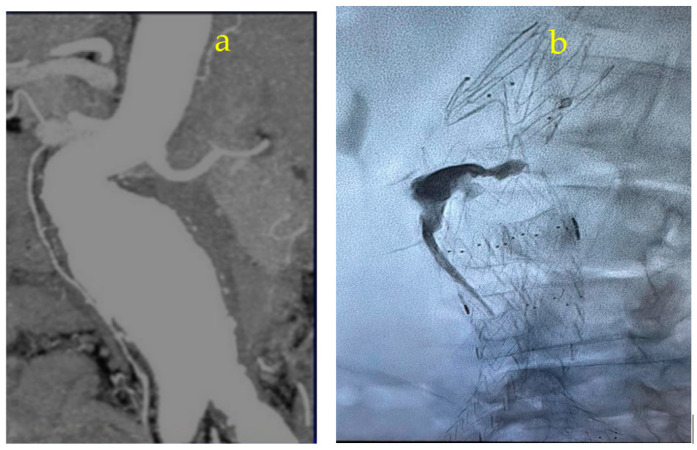
Pre-operative Tc MPR reconstruction (**a**), angiographic control with evidence of radiopacity of Onyx 18 (**b**).

## Data Availability

Not applicable.

## Appendix A

### Appendix A.1. Clinical Case 1: Right Renal Visceral Arteriovenous Malformation

A 54-year-old female patient goes to the emergency room for right-side pain. She has no signs of haematuria. We think of renal colic, but it is resistant to drugs and the symptoms persist. For this reason, a contrast-enhanced CT scan is carried out, which highlights the renal arteriovenous malformation (AVM) (Figure A1). The patient was taken to the operating room on the second day and underwent an AVM exclusion procedure.

Technique: Right percutaneous access, 6FR Destination 65 cm intruder, the 5F Simmons 100 cm catheter is positioned and control angiography is carried out with evidence of arteriovenous communication (Figure A2); the ASAHI Masters Parkway 2.7F 125 cm microcatheter is introduced coaxially and from this, the standard Penumbra Complex type coils no. 2 of 16 mm × 60 mm are released (Figure A3).

Post-procedural angiographic control is carried out (Figure A4) with evidence of exclusion of the AVM and correct positioning of the coils. Removal of devices and closure of access via percutaneous system.

### Appendix A.2. Clinical Case 2: Treatment of Endoleak Type II Inferior Mesenteric Artery

A 78-year-old patient, suffering from arterial hypertension and diabetes mellitus, following abdominal ultrasound colour Doppler examination for gallstones, with an occasional finding of AAA of 5.5 cm. The patient is invited to perform a contrast-enhanced CT exam, which highlights a subrenal abdominal aortic aneurysm and an IMA calibre of 5 mm.

Technique: Percutaneous access to the right common femoral artery, short 6FR intruder, intraoperative angiography is performed (Figure A5) with a 5 F 110 cm Pigtail catheter, confirming the presence of the aortorenal abdominal aorta aneurysm and the dilatation on the inferior mesenteric artery (IMA).

It is decided to treat the IMA, which could cause type II endoleak (ELTII) on a Terumo 0.035 guide. The catheter is changed by replacing the Pigtail with a Cobra 5F 100 cm catheter (Terumo) and a Progreat 2.4 F 110 cm microcatheter (Progreat™Microcatheter System Terumo, Terumo Italia S.r.l Via Paolo di Dono 73 00142 Rome, Italy) and the release of standard Penumbra Complex coils n°2 of 18 mm × 57 mm (Figure A6). The AAA is subsequently excluded with the placement of the Zenith Alpha™ Abdominal Endovascular Graft endoprosthesis (Cook Medical Europe Ltd., Limerick, Ireland). Final check (Figure A7) with the exclusion of the AMI and correct release of the spirals. Removal of devices and closure of access via percutaneous system.

### Appendix A.3. Clinical Case 3: Splenic Visceral Aneurysm

A 74-year-old patient suffering from arterial hypertension, COPD, undergoes abdominal ultrasound colour Doppler for renal control, with the occasional finding of a splenic aneurysm. CT with contrast medium was recommended and the patient was subsequently hospitalised for treatment to exclude the aneurysm.

Technique: Percutaneous access to the right common femoral artery, short 6 F introducer, on a Terumo 0.035 guide (Figure A8), position the Simmons 5 FR 125 cm catheter and carry out a control angiography with evidence of a splenic artery aneurysm (Figure A9). Subsequently, the REBAR microcatheter (terumo System) 2.8 F 135 cm is positioned in the coaxial line and the Concerto coils (detachable coil EV3) n°3 of 20 mm × 50 cm are released (Figure A10). Final angiographic check (Figure A11) and evidence of exclusion of aneurysm and correct positioning of the coils. Removal of devices and closure of access via percutaneous system.

### Appendix A.4. Clinical Case 4: Onyx Use in Endoleak Type 1 Excision Post EVAR ABI

A 78-year-old patient with regular follow-up for theoccasionalfinding of subrenal AAA; at last, an AngioTC examination of the calibre of aneurysm increased to 5.8 cm with evidence of angulated collar. It was decided to nominate the patient for surgery to exclude the aneurysm and the patient underwent exclusion of sub-renal abdominal aortic aneurysm (AAA) by EVAR ABI technique and the final peri-operative angiographic check showed endoleak type 1 (ELIA) and had to be corrected with Onyx embolising fluid.

Procedure: At the end of the procedure Medtronic Endurant II 23-16C124 (proximal stent graft) endoprosthesis placement by EVAR ABI technique from percutaneous access in DX femoral artery, Simmons 5 F 125 cm catheter is placed and using a Rebar 18 2.7F 137 cm microcatheter (Medtronic Rebar Reinforced Microcatheter, MEDTRONIC ITALIA S.P.A.Via Varesina, 162, 20156 Milano (MI), Italy), Onyx 18 is released to exclude the endoleak. The final angiographic check with the exclusion of endoleak and contrastographic/radiographic evidence of Onyxis shown in Figure A12.
